# Supplementary material for: Saccharomyces boulardii (CNCM I-745) improves intestinal damage in sepsis by remodeling intestinal flora structure
Source: Front Cell Infect Microbiol. 2025 Jul 31;15:1602792. doi: 10.3389/fcimb.2025.1602792 (PMC12350329; doi:10.3389/fcimb.2025.1602792)
Supplement: Supplementary file 1 [file DataSheet1.zip › summary/summary/7_Differential_analysis/1_diff_abundance/7_Species/Group.DW4_vs_CW4_vs_BW4_vs_AW4/Group.DW4_vs_CW4_vs_BW4_vs_AW4_diff_barplot.pdf]

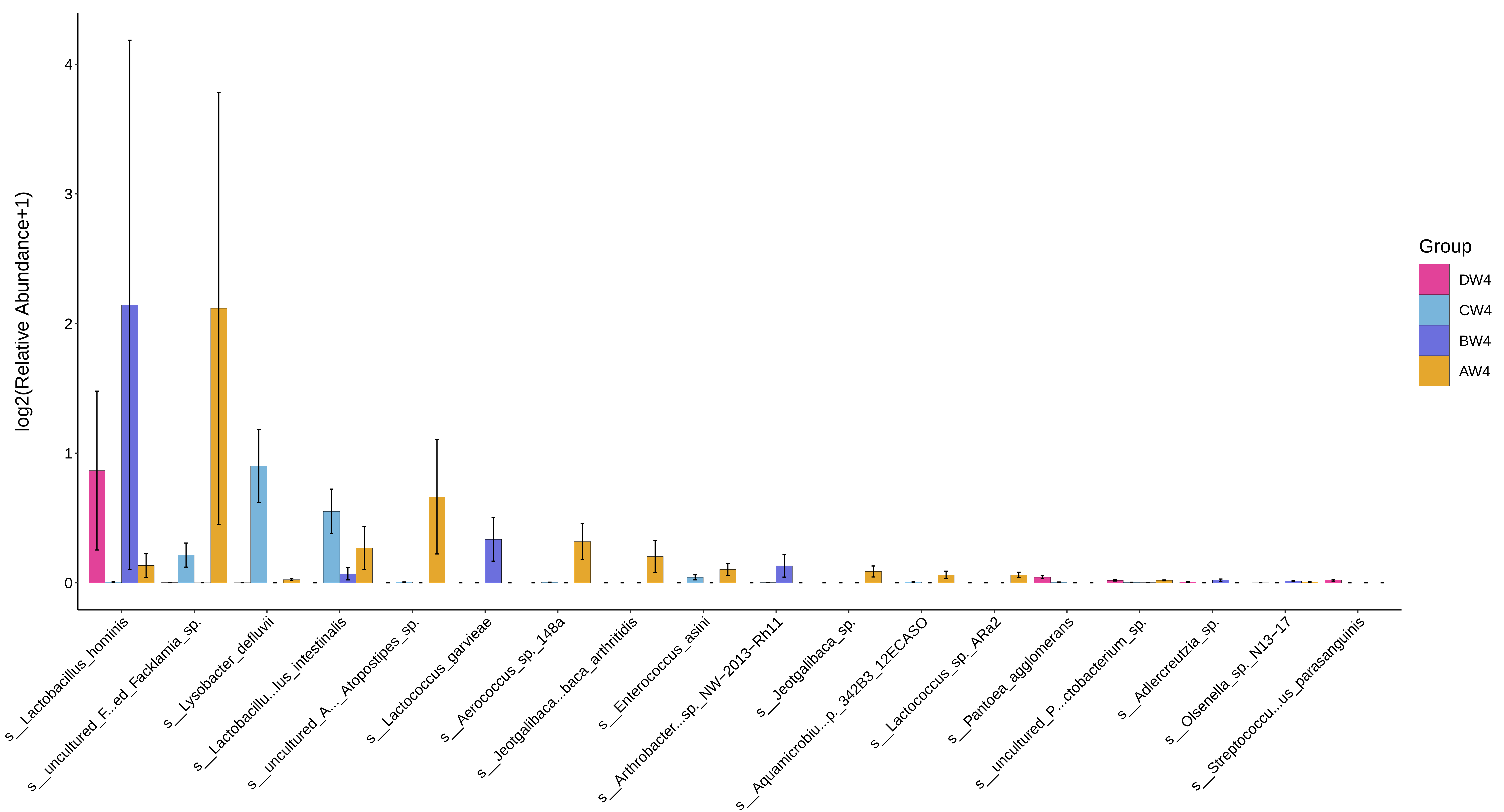
log2(Relative Abundance+1)
0
1
2
3
4
Group
DW4
CW4
BW4
AW4
s__Lactobacillus_hominis
s__uncultured_F...ed_Facklamia_sp.
s__Lysobacter_defluvii
s__Lactobacillu...lus_intestinalis
s__uncultured_A..._Atopostipes_sp.
s__Lactococcus_garvieae
s__Aerococcus_sp._148a
s__Jeotgalibaca...baca_arthritidis
s__Enterococcus_asini
s__Arthrobacter...sp._NW-2013-Rh11
s__Jeotgalibaca_sp.
s__Aquamicrobiu...p._342B3_12ECASO
s__Lactococcus_sp._ARa2
s__Pantoea_agglomerans
s__uncultured_P...ctobacterium_sp.
s__Adlercreutzia_sp.
s__Olsenella_sp._N13-17
s__Streptococcu...us_parasanguinis
